# 5-Aminolevulinic acid-guided resection improves the overall survival of patients with glioblastoma—a comparative cohort study of 343 patients

**DOI:** 10.1093/noajnl/vdab047

**Published:** 2021-03-26

**Authors:** Asfand Baig Mirza, Ioannis Christodoulides, Jose Pedro Lavrador, Anastasios Giamouriadis, Amisha Vastani, Timothy Boardman, Razna Ahmed, Irena Norman, Christopher Murphy, Sharmila Devi, Francesco Vergani, Richard Gullan, Ranjeev Bhangoo, Keyoumars Ashkan

**Affiliations:** 1 Department of Neurosurgery, King’s College Hospital NHS Foundation Trust, London, UK; 2 GKT School of Medical Education, King’s College London, London, UK

**Keywords:** 5-aminolevulinic acid, glioblastoma, overall survival, performance status, resection

## Abstract

**Background:**

5-Aminolevulic acid-guided surgery (5-ALA-GS) improves the extent of resection (EoR) and progression-free survival in patients with glioblastoma multiforme (GBM).

**Methods:**

A single-center retrospective cohort study of adult patients with GBM who had surgical resection between 2013 and 2019, 5-ALA guided versus a non-5-ALA cohort. The primary outcome was the overall survival (OS). Secondary outcomes were EoR, performance status (PS), and new focal neurological deficit.

**Results:**

Three hundred and forty-three patients were included: 253 patients in 5-ALA-GS group and 90 patients in the non-5-ALA-GS group. The OS (17.47 vs 10.63 months, *P* < .0001), postoperative PS (*P* < .0001), PS at 6 months (*P* = .002), new focal neurological deficit (23.3% vs 44.9%, *P* < .0001), and radiological EoR (gross total resection [GTR]—47.4% vs 22.9%, *P* < .0001) were significantly better in the 5-ALA-GS group compared to non-5-ALA-GS group. In multivariate analysis, use of 5-ALA (*P* = .003) and MGMT promoter methylation (*P* = .001) were significantly related with a better OS. In patients with radiological GTR, OS was also significantly better (*P* < .0001) in the 5-ALA-GS group compared to the non-5-ALA-GS group.

**Conclusions:**

5-ALA-GS is associated with a significant improvement in the OS, PS after surgery and at 6 months, larger EoR, and fewer new motor deficits in patients with GBM.

**Key Points**

- 5-ALA-guided resection improves overall survival of WHO grade 4 glioblastoma patients when compared with non-5-ALA-guided surgery.
- 5-ALA-guided surgery improves EoR and PFS when compared with non-5-ALA-guided surgery in WHO grade 4 glioblastoma patients.

**Importance of the Study**

While 5-aminolevulinic acid (5-ALA) use is now a common method of fluorescence-guided surgery as it has been shown to improve the rate of resection and progression-free survival compared to white light, mixed evidence has been published on longer-term overall survival mainly derived from a highly selected cohort of patients. Of the literature that currently exists, most are generally small-scale studies, creating the potential for larger variation and bias of results. We believe our study presents data from the largest single-center cohort of patients operated on under 5-ALA to date. With this number of patients, we hope we can reduce the risk of bias as a result of small sample sizes and thus provide more reputable data.

The main goals of surgical treatment in glioblastoma (GBM) patients are maximal safe resection while preserving the quality of life and providing an accurate histopathological and molecular diagnosis that will help guiding the adjuvant treatment. This would ideally translate in the resection of the radiologically defined lesion, a situation that is difficult to reproduce in the intraoperative setting, due to the surgeon’s limitation of confidently distinguishing the brain–tumor interface under conventional white-light microscopy.^1^ Several intraoperative techniques, including neuro-navigation, intraoperative MRI, and ultrasound, have been developed and applied, in an attempt to encourage and ensure greater tumor resection, although each technique has its own limitations.

5-Aminolevulinic acid (5-ALA) is the cornerstone of fluorescence-guided brain surgery. It is a precursor of the protoporphyrin IX molecule, which in high concentrations allows fluorescence.^2–4^ This oral chemical agent that is administered preoperatively demonstrates high selective accumulation within the pathological tissue, in particular, GBMs.^5–7^ As a result, tumor tissue becomes fluorescent under blue-light microscopy. This fluorescence is independent of brain volume changes and brain shift, providing truly real-time guidance to the surgeon. Integration of this adjunct with preoperative and intraoperative mapping has revolutionized the surgical approach to GBMs (Figure 1).

**Figure 1.**
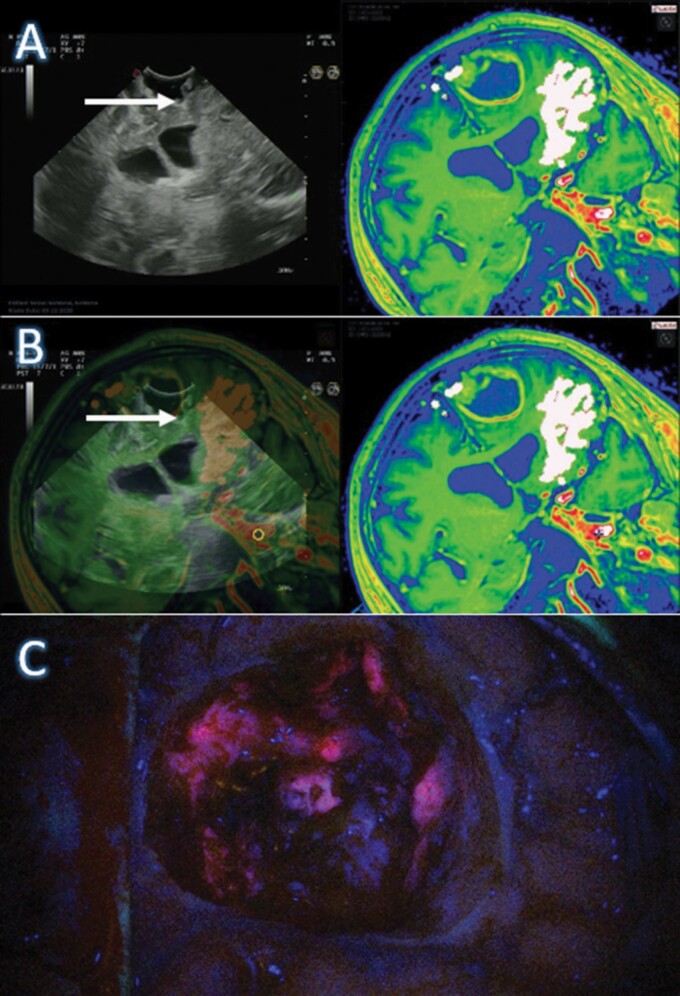
Integrated intraoperative ultrasound (US) and MRI (A and B) and 5-ALA in the surgical cavity (C) global assessment: fusion of MRI with integrated preoperative dissection of the fronto-aslant tract, intraoperative US, and 5-ALA to assess the extent of resection. *White Arrow*—contrast-enhancing tumor identified in the US-fMRI fusion and confirmed with 5-ALA fluorescence.

Intraoperative fluorescence allows identification of pathological tissue and a clearer visualization of the brain–tumor interface, allowing the neurosurgeon to extend the resection toward or beyond the contrast-enhancement areas on MRI (Figure 1). Indeed, when compared to conventional white-light resection, 5-ALA use has demonstrated the improved extent of resection (EoR; 36% in white-light compared to 65% with 5-ALA), which translated into a near twice improvement in the 6-month progression-free survival (PFS; 41% vs 21%) and a more than 3-month increase in the overall survival rates with no worsening of neurological deficits.^7–16^

Nevertheless, the majority of the data on 5-ALA-guided surgery (5-ALA-GS) stem from small case series with short follow-up periods, making any recommendations less robust. In this report, we present our institution’s experience with 5-ALA-assisted resection of GBMs, which to our knowledge is the largest to date.

## Methods

This is a single-center retrospective cohort study between January 2013 and January 2019 of patients operated with 5-ALA for GBMs. The inclusion criteria were ≥18 years old, 5-ALA-GS, pathology consistent with WHO grade 4 GBM, and consent form signed for the surgical procedure. The exclusion criteria were non-glial tumor, non-WHO grade 4, surgical biopsies, and incomplete medical records.

5-ALA was administered via oral route with a dosage of 20 mg/kg to a maximal dose of 1500 mg per patient. The ideal time of administration was 2–4 h prior to surgery (even though administration outside this time frame was not considered an exclusion criterion for this study). Intraoperatively, 2 different microscopes were used during the 5-ALA-assisted procedures—PENTERO 900 and KINEVO—with the BLUE 400 Filter from ZEISS Medical Technology.

Demographic and clinical data were collected from patients’ medical records. The primary outcome was to assess the impact of 5-ALA-GS on the overall survival in patients diagnosed with GBM. The secondary outcomes were its impact on the postoperative performance status (PS), PS at 6 months after surgery, the EoR, new focal neurological deficit after surgery, and length of hospital stay. Gross total resection (GTR) was defined as no residual contrast enhancement detected on the postoperative MRI scan performed within 72 h of surgery while subtotal resection (STR) was defined as residual contrast enhancement.^17^ The results were compared with a cohort of patients treated by the same team in our institution, prior to implementation of a regular program of 5-ALA-GS for GBMs (January 2009–January 2013). Similar inclusion and exclusion criteria apart from 5-ALA-GS were applicable for the control cohort.

A literature review of the case series published in the last 10 years was also performed. We have excluded those where the survival outcomes were not reported as per EoR (GTR vs STR); studies were divided according to the intraoperative use of 5-ALA.

Regarding ethical approval, all procedures performed in studies involving human participants were in accordance with the ethical standards of our institution and with the 1964 Helsinki declaration and its later amendments or comparable ethical standards. For this type of study where data were collected during routine clinical care of patients, formal consent is not required. The use of 5-ALA was approved by our institution’s New and Novel Procedures Committee.

STATA 13.0 statistical software was used for the statistical analysis. Chi-square, *T*-test, and regression analysis (multinomial, ordered, and logistic) were performed to investigate the relationship between the variables considered. Multinomial Cox-Hazard statistics were used for survival analysis. A *P* value less than .05 was considered statistically significant. An adjusted regression model for confounding factors, age, gender, preoperative PS, use of intraoperative neurophysiological monitoring (IONM), *isocitrate dehydrogenase* (IDH) mutation status, *O-6-methylguanine-DNA methyltransferase* (MGMT) methylation status, radiological EoR, postoperative PS, PS at 6 months, adjuvant treatment (radiotherapy and chemotherapy), and the use of 5-ALA, was performed.

## Results

### Patient Demographics

Three hundred and forty-three patients fulfilled the inclusion criteria: 253 patients had 5-ALA-GS and 90 patients had non-5-ALA-GS. Both groups had a predominantly male gender, similar tumor locations, a comparable mix of first and redo surgery, and a similar age distribution (5-ALA-GS: 56.69 ± 0.75 vs non-5-ALA-GS: 54.43 ± 1.73, *P* = .234). The 5-ALA-GS group had lower preoperative PS (*P* < .0001), postoperative PS (*P <* .0001), PS at 6 months (*P =* .002), and higher utilization of intraoperative neuromonitoring (31.8% vs 12.3%, *P* < .0001), reflecting the gradual change in our practice over time. Both groups had a similar distribution of ATRX and IDH mutations. The 5-ALA-GS group had a lower rate of MGMT promoter methylation (50.6% vs 68.3%, *P* = .015; Table 1).

**Table 1.** Overall Characteristics of Non-5-ALA-GS and the 5-ALA-GS Groups

	Non-5-ALA-GS Group (*n* = 90)	5-ALA-GS Group (*n* = 253)	*P*
Gender			
Male	57	174	
Female	33	79	.406
Age (years)	54.43 ± 1.73	56.69 ± 0.75	.234
Surgical resection			
First craniotomy	68	211	
Redo craniotomy	22	42	
Preoperative PS			
0	32	128	
1	27	95	
2	21	23	
3	4	5	
4	6	2	<.0001
Postoperative PS			<.0001
0	28	94	
1	30	120	
2	11	21	
3	13	13	
4	8	5	
PS at 6 months			.002
0	14	37	
1	17	67	
2	4	25	
3	6	19	
4	9	5	
5	13^a^	12^b^	
Location			
Frontal lobe	30	83	.830
Temporal lobe	32	92	(base outcome)
Parietal lobe	21	48	.285
Occipital lobe	5	26	.390
Others	2	4	.989
Intraoperative neuromonitoring	7 (7.8%)	77 (30.4%)	<.0001
Molecular markers			
ATRX	37 (88.1%)^c^	167 (90.6%)^h^	.624
IDH	5 (7.5%)^d^	19 (7.7%)^i^	.981
MGMT	41 (68.3%)^e^	120 (50.6%)^j^	.015
Chemotherapy	43 (74.1%)^f^	174 (95.1%)^k^	<.0001
Radiotherapy	48 (76.2%)^g^	181 (96.3%)^l^	<.0001

5-ALA-GS, 5-aminolevulinic acid-guided surgery; PS, performance status; ATRX, *alpha-thalassemia x-linked intellectual disability*; IDH, *isocitrate dehydrogenase*; MGMT, *O-6-methylguanine-DNA methyltransferase*.

Values indicated in bold are statistically significant (P < 0.05).

^a^27 patients lost for follow-up.

^b^88 patients lost for follow-up.

^c^48 patients with no data.

^d^24 patients with no data.

^e^30 patients with no data.

^f^32 patients with no data.

^g^27 patients with no data.

^h^72 patients with no data.

^i^5 patients with no data.

^j^16patients with no data.

^k^70 patients with no data.

^l^65 patients with no data.

The time interval between administration of 5-ALA and the start of the surgery did not significantly affect the EOR (*P* = .102). The use of IONM was related to larger EoR in the 5-ALA-GS group (*P* = .042) but not in the non-5-ALA-GS group (*P* = .710).

Chemotherapy regimen and radiotherapy doses were the same for the historical control group and the 5-ALA group. These were based on the STUPP protocol^18^ for first presentation cases and consisted of concomitant 6 weeks of radiotherapy with 60 Gy combined with temozolomide chemotherapy followed by 6 cycles of adjuvant temozolomide chemotherapy. For patients with recurrent GBM, procarbazine, lomustine, and vincristine combination chemotherapy was the regimen of choice.

### Primary and Secondary Outcomes

**Figure 2.**
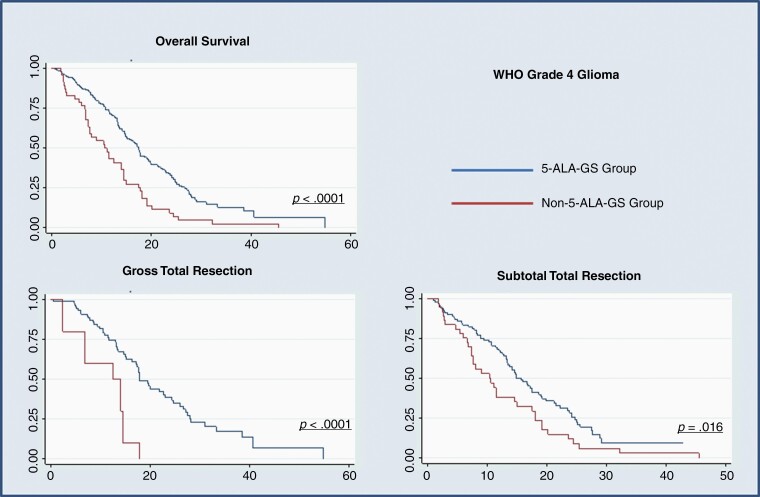
Kaplan–Meier curves of our results. 5-ALA-GS, 5-aminolevulinic acid-guided surgery.

The overall survival was significantly better with 5-ALA-GS (17.47 vs 10.63 months, *P* < .0001). Additionally, postoperative PS (*P* < .0001), PS at 6 months after surgery (*P* = .002), new temporary focal neurological deficit (23.3% vs 44.9%, *P* < .0001), and the radiological EoR (GTR—47.4% vs 22.9%, *P* < .0001) were significantly better in the 5-ALA-GS group (Table 2). Furthermore, significantly more patients in the 5-ALA-GS group were able to complete postoperative chemotherapy and radiotherapy (92.8% vs 79.8%, *P* = .001; 93.9% vs 83.5%, *P* = .004, respectively; Figure 2 and Table 2).

**Table 2.** Outcomes and Risk Factor Analysis in Glioblastomas

Primary Outcome			
	HR	95% CI	*P*
Overall survival	2.07 ± 0.36	1.47–2.93	<.0001 (Cox)
Secondary Outcomes			
	Coef.	95% CI	*P*
Postoperative PS	0.43 ± 0.12	0.19–0.687	<.0001 (logit)
New focal neurological deficit	1.26 ± 0.29	0.69–1.82	<.0001
EoR	1.11 ± 0.29	0.54–1.68	<.0001
PS at 6 months of FU	0.28 ± 0.09	0.10–0.46	.002
Risk Factors for Overall Survival (Unadjusted)			
	HR	95% CI	*P*
Gender	0.87 ± 0.14	0.64–1.19	.386
Age	1.01 ± 0.01	1.00–1.03	.028
Preoperative PS	1.33 ± 0.11	1.13–1.56	<.0001
IONM	0.54 ± 0.11	0.37–0.81	.002
IDH	0.66 ± 0.22	0.35–1.26	.213
MGMT	0.42 ± 0.07	0.31–0.59	<.0001
Radiological extent of resection	1.42 ± 0.22	1.04–1.93	.028
Postoperative PS	1.40 ± 0.11	1.20–1.64	<.0001
PS at 6 months of FU	1.53 ± 0.08	1.37–1.70	<.0001
New temporary motor deficit	1.42 ± 0.28	0.96–2.10	.082
Radiotherapy	0.20 ± 0.06	0.11–0.37	<.0001
Chemotherapy	0.04 ± 0.1	0.02–0.08	<.0001
Non-5-ALA-assisted surgery	2.07 ± 0.36	1.47–2.93	<.0001
Risk Factors for Overall Survival (Adjusted)			
	HR	95% CI	*P*
Gender	0.77 ± 0.21	0.45–1.32	.347
Age	1.01 ± 0.13	0.98–1.03	.605
Preoperative PS	0.81 ± 0.16	0.55–1.21	.319
IONM	0.72 ± 0.22	0.40–1.31	.281
IDH	1.54 ± 0.89	0.500–4.75	.451
MGMT	0.39 ± 0.11	0.22–0.68	.001
Radiological extent of resection	1.02 ± 0.28	0.60–1.74	.942
Postoperative PS	1.34 ± 0.27	0.91–1.98	.144
PS at 6 months of FU	1.19 ± 0.11	0.99–1.42	.052
Radiotherapy	0.78 ± 0.74	0.12–5.00	.790
Chemotherapy	0.19 ± 0.18	0.03–1.30	.091
Non-5-ALA-GS	2.95 ± 1.09	1.43–6.09	.003

5-ALA-GS, 5-aminolevulinic acid-guided surgery; PS, performance status; EoR, extent of resection; FU, follow-up; IONM, intraoperative neurophysiological monitoring; IDH, *isocitrate dehydrogenase*; MGMT, *O-6-methylguanine-DNA methyltransferase*.

Values indicated in bold are statistically significant (P < 0.05).

### Risk Factor Analysis—Adjusted and Unadjusted Analysis

Older age, higher preoperative PS, nonuse of IONM, unmethylated MGMT promotor, STR, higher postoperative PS, higher PS at 6 months, a new temporary motor deficit, lack of adjuvant treatment (radiotherapy and/or chemotherapy), and non-5-ALA-GS were related with worse overall survival (Table 2). When the model was adjusted, MGMT promoter methylation and 5-ALA-GS emerged as the only factors related to an improvement in the overall survival (Table 2).

### Subgroup Analysis—GTR Versus STR

The 5-ALA-GS group had a better overall survival both for those in whom GTR was achieved (17.77 months vs 14.03 months, *P* < .0001) and in those undergoing STR (15.67 months vs. 10.4 months, *P* = .016). Postoperative PS was also significantly better in the *5-ALA-GS group* both for those undergoing GTR (*P* = .014) and those with STR (.029) although new transitory focal deficit was less common in STR compared to the GTR group (18% vs 45%, *P* < .0001; Supplementary Material 1). Postoperative FLAIR images were only available in a limited number of patients who underwent GTR, 57 in the 5-ALA-GS group and 3 in the historical controls. Within clear constraints of such small numbers, the cavity of resection was noted to include at least some of the FLAIR volume, beyond the contrast enhancement, in 80% when surgery was guided by 5-ALA versus 30% in the control group.

## Discussion

The use of 5-ALA received FDA approval in 2017 and became routinely reimbursed in the United Kingdom in 2019.^19^ In our center, however, 5-ALA-assisted surgery was introduced on an ad-hoc basis in 2010 and then became part of the routine practice of managing GBMs from 2014. Our data here show that our practice of 5-ALA-GS has been associated with significant improvement in overall survival, EOR, postoperative PS, PS at 6 months after surgery, and completion of adjuvant therapy in patients with WHO grade 4 GBMs. Moreover, the adjusted regression model for confounding factors excluded other technical adjuncts (such as the use of IONM) as the reason for the improvement in survival. The biological signature of the tumors (MGMT) and the use of 5-ALA were shown to be the only factors related to survival in the multifactorial analysis.

Although the impact of 5-ALA on enhancing the intraoperative visualization of tumors, improving the EOR, and PFS has been well established in the literature, data on its influence on overall survival are limited.^16,20,21^ Our study, the largest series thus far on 5-ALA-GS, indicated improved overall survival in patients with GBMs undergoing 5-ALA-GS even after adjustment for other factors known to influence survival in these patients. Table 3 summarizes the surgical case series published in the last 10 years on the impact of surgery on the overall survival of patients with grade 4 gliomas.^12,22–40^ These indicate a general trend toward an increase in the utilization of 5-ALA-GS in GBMs with an associated increase in EoR and a higher percentage of patients undergoing GTR. The patient numbers in these series, however, remain small with a median of 33 (range: 13–103), often with no adequate control group, limiting the conclusions on PFS and especially overall survival.

**Table 3.** Literature Review of the Surgical Case Series Published With Non-5-ALA-GS and 5-ALA-GS for WHO Grade 4 Gliomas in the Last 10 Years in Patients Treated With Stupp Protocol (enrollment after 2006)

Author (Year)	Years Included	No. of Patients	Median/Mean Age (Years)	5-ALA	Pre-Op KPS	Tumor Type (WHO Grade)	Surgical Outcome	Post-Op KPS	Follow-up KPS	Progression-Free Survival (Months)	Overall Survival (Months)
*Nonfluorescence-Guided Surgery Literature*											
Dobran et al. (2019)^22^	2012–2015	40	60	N	64	GBM (IV)	GTR: 25	72	70	N/A	GTR: 9
							STR: 15				STR: 6
Guler et al. (2019)^23^	2007–2014	126	55	N	N/A	GBM (IV)	GTR: 31	70–80: 51	N/A	N/A	GTR: 23.6
							STR: 85	90–100: 75			STR/Biopsy: 14.1
							Biopsy: 10				
Dayani et al. (2018)^24^	2004–2014	39	57.8	N	80	bGBM (IV)	Resection: 14 (median EOR: 83%)	80	N/A	N/A	EOR >86%: 17.7
											EOR <86%: 2.4
							Biopsy: 25				
											Biopsy: 2.5
Chan et al. (2017)^25^	2010–2012	42	55.1	N	N/A	GBM (IV)	GTR: 15	N/A	N/A	N/A	GTR: 16
							STR: 18				STR: 13
							Biopsy: 9				
											Biopsy: 4.5
Harris et al. (2017)^26^	2006–2016	108	78.7	N	N/A	GBM (IV)	GTR: 40	N/A	N/A	N/A	GTR: 12
							STR/Biopsy: 30				STR/Biopsy: 6.7
Guden et al. (2016)^27^	2006–2015	92	59	N	<70: 17	GBM (IV)	GTR: 56	N/A	N/A	GTR: 29.4	GTR: 30.8
							STR: 24			STR: 29.8	STR: 24.7
					>70: 74						
							Biopsy: 12				
Ringel et al. (2016)^28^	2006–2010	503	57	N	90	Recurrent GBM (IV)	GTR: 238	90	N/A	N/A	GTR–GTR: 13.6
							STR: 170				
											GTR–STR: 9.2
							PR: 39				
											STR–GTR: 12.2
											STR–STR: 10.2
Hrabalek et al. (2015)^29^	2007–2009	22 (GTR)	62.5	N	80	GBM (IV)	GTR: 3	80	N/A	N/A	GTR: 11.3
							NTR: 6				NTR: 17.1
											STR: 7.8
							STR: 7				PR: 9.7
							PR: 6				
Qin et al. (2015)^30^	2010–2014	816	—	N	<80: 206	748× Primary GBM (IV), 68× Secondary GBM (IV)	GTR: 209	N/A	N/A	GTR: 12.5	GTR: 19.9
							STR: 607			STR: 8	STR: 14.8
					≥80: 610						
Lombardi et al. (2015)^31^	2007–2014	237	71	N	N/A	GBM (IV)	GTR: 174	N/A	N/A	N/A	GTR: 17.7
							STR: 63				STR: 16.1
Chaichana et al. (2014)^32^	2007–2011	259	59.6	N	N/A	GBM (IV)	Unspecified, 81% average extent of resection	N/A	N/A	N/A	>70%: 14.4
											≤70%: 10.5
Hong et al. (2013)^33^	2006–2010	42	A:60, B:56.5	N	N/A	GBM (IV)	A: GTR: 8 STR: 2	A: <80: 0 ≥80: 10	N/A	N/A	GTR: 16
											STR: 14
								B: <80: 14 ≥80: 18			
							B: GTR: 26 STR: 6				
Yabroff et al. (2012)^34^	2006	1202	—	N	N/A	1170× Glioblastoma (IV)	GTR: 461	N/A	N/A	N/A	GTR: 20
							PR: 314				PR: 13
											Biopsy: 14
						11× GCGBM (IV)					
							Biopsy: 29				No surgery: 10
						21× Gliosarcoma (IV)					
							Other: 120				
							No surgery: 275				
							Unknown: 3				
*Fluorescence-Guided Surgery Literature*											
Della Puppa et al. (2017)^35^	2009–2013	20	55.3	20× 5-ALA + BCNU wafers	100	GBM (IV)	GTR: 100%	Not stated	Not stated	11	22
Hauser et al. (2016)^36^	2009–2011	13	60.5	Y	N/A	GBM (IV)	GTR: 10	N/A	N/A	N/A	GTR: 18.9
							STR: 3				STR: 8.3
Coburger et al. (2015)^37^	2008–2012	33	57	5-ALA + iMRI	N/A	GBM (IV)	GTR 100%	Not stated	Not stated	6	17
Kim et al. (2014)^38^	2009–2011	40	51	5-ALA	<80: 13	GBM (IV)	GTR: 80%	82.7	84.7	18	GTR: 24
							STR: 20%				STR: 13
					80–90: 3						
					90–100: 24						
Aldave et al. (2013)^12^	2007–2011	52	58.7	Y	<80: 20	GBM (IV)	GTR: 100%	N/A	N/A	N/A	Nonresidual fluorescence = 27m, 17.5m with residual fluorescence
					≥80: 32						
Schucht et al. (2012)^39^	2008–2010	103	62	Y	83	GBM (IV)	GTR: 53	84	N/A	N/A	GTR: 16.7
											STR: 11.8
							STR: 16				
							Biopsy: 34				
Diez Valle et al. (2011)^40^	2007–2009	28	58	Y	N/A	GBM (IV)	GTR: 23	70 (mean)	Not Stated	GTR: 8.5	GTR: 10.6
							STR: 5			STR: 10.7	STR: 13

EoR, extent of resection; GBM, glioblastoma multiforme; bGBM, butterfly glioblastoma multiforme; GTR, gross total resection; STR, subtotal resection.

**Figure 3.**
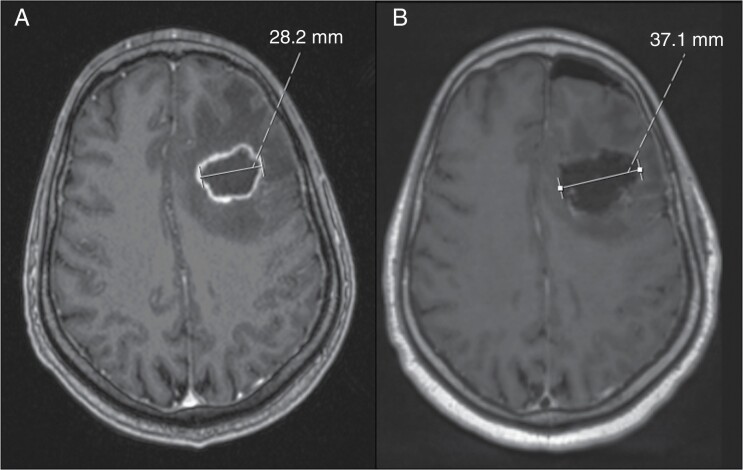
(A) Pre- and (B) postoperative axial contrast-enhanced MRI showing the size of the resection cavity beyond the limits of contrast enhancement.

5-ALA-GS was found to improve the overall survival in both GTR and STR cohorts. While the improvement in overall survival of patients with WHO grade 4 gliomas in the 5-ALA-GS group compared to the controls might be attributable to improved EoR in patients with STR, the better overall survival in those with GTR in the 5-ALA-GS group is less readily explained. In part this may reflect changes in the overall care of patients during our relatively long study period, given the sequential nature of the recruitment. In part though, the observed difference might highlight the challenges in how GTR is defined. Radiologically for GBMs this has been based on complete resection of enhancing tumor. Literature, however, is emerging to show that the 5-ALA-dependent fluorescent tissue tends to extend beyond the limits of contrast-enhancing tumor on the MRI.^41,42^ Indeed, Yamada et al.^43^ reported that the GTR achieved with the use of 5-ALA, in terms of the relationship between the size of the surgical cavity versus the size of the preoperative contrast-enhancing tumor, was substantially different in patients undergoing combined 5-ALA/intraoperative MRI surgery versus those with intraoperative MRI alone. In fact, more recently, better outcomes in GBMs have been reported when the resection was extended beyond the contrast-enhancement limits to incorporate the FLAIR volume.^44,45^ In our cohort, analysis of the limited number of postoperative FLAIR images available, also signaled in favor of a larger resection cavity beyond the contrast enhancement when achieving GTR with 5-ALA-GS. Thus, 5-ALA-GS might result in a “more” maximal resection than that assessed based purely on contrast enhancement on MRI (Figure 3). Large prospective studies with detailed volumetric MRI analysis are, however, required to fully explore this concept.

The impact of time between 5-ALA administration and the surgery on the outcomes was assessed. This is particularly significant in public health systems where urgent and emergent admissions can be responsible for delays in surgery. Although others have found a significant correlation between the EoR and the time interval between 5-ALA administration and onset of surgery,^46^ no such correlation was identified in our patients undergoing craniotomy with a mean time of 4 h and 25 min between administration of 5-ALA and surgery.

The ultimate goal of GBM treatment is the improvement or preservation of quality of life. A good PS is crucial for tolerance, ability to complete, and thus maximal benefit from adjuvant treatment.^47–49^ 5-ALA-GS was associated with a better PS at 6 months after surgery in both unadjusted and adjusted analysis and a higher probability of patients completing their adjuvant treatment. This further supports the use of this adjunct in GBM surgery, not only for its direct effect on EoR and survival but also for its impact on PS and facilitating adjuvant therapies.

## Limitations

Our study is prone to the limitations of a retrospective series spanning a long recruitment period. Data on the molecular tumor markers, particularly IDH, 1p19q, and MGMT promoter methylation, were not available for all patients. These became routinely available since the publication of the new Classification of Tumors of the Central Nervous System in 2016 but were not systematically available before that date.^50^ Where historical tumor specimens were available, we carried out the assessments retrospectively but this was not the case for all.

Our control group was historical and therefore prone to the biases related to the evolution of our practice. For example, more patients in the 5-ALA group received adjuvant therapy than those in the control cohort which may have contributed to the differences seen in the survival outcomes although not in EOR, postoperative neurological deficit, or PS. The surgical learning curve may have also played a role here given that the control group was operated in the years prior to the 5-ALA group. Nonetheless, the surgical team had over two decades of experience beyond the steep section of such a learning curve and proficient in surgical techniques for tumor resection. Furthermore, the 5-ALA-GS technique itself is open to a learning curve that needs to be borne in mind. Nonetheless, by adjusting our analytic model, we addressed these limitations where possible, showing the MGMT status of the tumor and 5-ALA-GS as the only factors related to an improvement in the overall survival, rather than, for example, the use of IONM.

Our observation of improved survival in the 5-ALA-GS group, even in those undergoing GTR compared to controls, and the possible contribution of 5-ALA-aided resection beyond the contrast-enhanced tumor on MRI, requires further investigation in prospective volumetric MRI studies.

## Conclusion

Within limitations of a retrospective study, the data from this largest series on 5-ALA-GS in patients with GBM show significantly better overall survival, PFS, EOR, and postoperative PS with the use of 5-ALA compared to controls.

## Supplementary Material

vdab047_suppl_Supplementary_Materials

## Notes

**Conflict of interest statement**. All authors certify that they have no affiliations with or involvement in any organization or entity with any financial interest (such as honoraria; educational grants; participation in speakers’ bureaus; membership, employment, consultancies, stock ownership, or other equity interest; and expert testimony or patent-licensing arrangements) or nonfinancial interest (such as personal or professional relationships, affiliations, knowledge, or beliefs) in the subject matter or materials discussed in this manuscript.

## Notes

**Authorship Statement.** Conceptualization ideas: K.A. and J.P.L.; methodology: K.A., G.A., and J.P.L.; formal analysis: J.P.L.; investigation—data curation: R.A., A.B.M., T.B., Y.C., S.D., R.N., C.M., and A.V.; literature review: Y.C., A.B.M., T.B., and A.V.; writing: K.A., Y.C., J.P.L., and A.B.M.; visualization preparation: A.B.M. and J.P.L.; reviewing and editing: K.A., A.B.M., R.B., R.G., J.P.L., and F.V.; supervision oversight: K.A., R.B., R.G., and F.V.
