# Supplementary material for: 5-Aminolevulinic acid-guided resection improves the overall survival of patients with glioblastoma—a comparative cohort study of 343 patients
Source: Neurooncol Adv. 2021 Mar 26;3(1):vdab047. doi: 10.1093/noajnl/vdab047 (PMC8193902; doi:10.1093/noajnl/vdab047)
Supplement: vdab047_suppl_Supplementary_Materials [file vdab047_suppl_supplementary_materials.docx]

| Gross Total Resection | | | |
| --- | --- | --- | --- |
|  | **HR** | **95%CI** | **p value** |
| Overall Survival | 3.78±1.39 | [1.84 – 7.76] | <0.0001 |
|  | **Coef.** | **95%CI** | **p value** |
| Post-Operative PS | 0.66±0.27 | [0.13 – 1.18] | 0.014 |
| New Focal Neurological Deficit | 0.35±0.69 | [-1.00 – 1.70] | 0.612 |
| PS at 6 months of FU | 0.18±0.20 | [-0.20 – 0.57] | 0.353 |
|  |  |  |  |
| Subtotal Resection | | | |
|  | **HR** | **95%CI** | **p value** |
| Overall Survival | 1.65±0.35 | [1.10 – 2.49] | 0.016 |
|  | **Coef.** | **95%CI** | **p value** |
| Post-Operative PS | 0.31±0.14 | [0.03 – 0.58] | 0.029 |
| New Focal Neurological Deficit | 1.32±0.34 | [0.65 – 1.99] | <0.0001 |
| PS at 6 months of FU | 0.30±0.11 | [0.09 – 0.52] | 0.006 |

**Supplementary Material 1** – Analysis according to the Extent of Resection in patients diagnosed with glioblastomas (5-ALA-GS Group *versus* Non-5-ALA-GS Group). PS = Performance Status; FU = Follow-up.
